# Clinical evaluation of an over-the-counter hearing aid (TEO First®) in elderly patients suffering of mild to moderate hearing loss

**DOI:** 10.1186/s12877-016-0304-4

**Published:** 2016-07-09

**Authors:** Guillaume Sacco, Sébastien Gonfrier, Bernard Teboul, Ivan Gahide, Fredéric Prate, Mathilde Demory-Zory, Jean-Michel Turpin, Claire Vuagnoux, Philippe Genovese, Stéphane Schneider, Olivier Guérin, Nicolas Guevara

**Affiliations:** Center for Healthcare Innovation and Uses (CIU-santé), University Hospital of Nice, Cimiez Hospital, Pavillon Mossa 4ème étage aile Sud, 4 av Reine Victoria, 06000 Nice, France; CoBTeK Cognition Behaviour Technology EA 7276, Research Center Edmond and Lily Safra, Nice Sophia-Antipolis University, Nice, France; Rehabilitation Unit, Department of Geriatrics, University Hospital of Nice, Cimiez Hospital, Nice, France; Long Care and Nursing Home Unit, Department of Geriatrics, University Hospital of Nice, Cimiez Hospital, Nice, France; ENT Unit, University Hospital of Nice, University institute of face and neck (IUFC), Nice, France; Acute Geriatrics Unit, Department of Geriatrics, University Hospital of Nice, Cimiez Hospital, Nice, France

**Keywords:** OTC Hearing aid, Elderly, Hearing rehabilitation

## Abstract

**Background:**

Presbycusis has a direct influence on autonomy of the elderly but hearing aids lack of affordability. Moreover a recent review demonstrate that electroacoustic characteristics of OTC hearing aids were generally not suitable for the elderly people. In our study, we assessed the clinical value of a new over-the-counter (OTC) hearing aid device (TEO First®) in the elderly.

**Method:**

This prospective monocentric open label study included patients over 60 years of age with a mild to moderate presbycusis. Patients were assessed with acceptable noise level test (ANL), pure tone (PTA) and speech (SA) audiometry in silent and noisy environment, with and without TEO First®. A Glasgow Hearing Aid Benefit Profile, acceptability and satisfaction surveys were completed after one month of using the device.

**Results:**

Thirty one patients were included. There was an improvement of hearing with TEO First® in silence (SA: +39.2 %, *p* < 0.01; PTA: -9.04 dB, *p* < 0.01) or in noise (SA +47.7 %, *p* < 0.01; PTA: -5.23 dB, *p* < 0.05). After one month of use of the device, quality of life has improved with regards to the following parameters: decrease of perceived hearing difficulties during conversation without background noise (-9.6 % *p* = 0.018), in conversation with several people (-16.2 % *p* = 0.0076), decrease of negative emotions while watching TV (-18.5 % *p* = 0.011), during conversation without background noise (-16.5 % *p* = 0.0024), during conversation in noisy background (-17.1 % *p* = 0.027) and during conversation with several people (-20 % *p* = 0.014). The acceptability of the device was low to moderate.

**Conclusion:**

TEO First® is an effective OTC hearing aid that improves the patient’s quality of life.

**Trial registration:**

Current Controlled Trials NCT01815788

## Background

Hearing loss affects 8 to 10 % of the European population [1] and its first etiology is presbycusis [2]. Presbycusis is a sensorineural hearing loss for which an early prosthetic and non-prosthetic treatment is recommended [2]. External hearing aid constitutes the first-in-line prosthetic treatment in perceptive hearing loss, thus including mild and moderate presbycusis.

Frailty could be defined as a reversible state associated with subtle physical, psychological, and social impairments that could lead to loss of autonomy. The evaluation of hearing capabilities is a part of frailty assessment. Indeed, hearing loss widely contributes to loss of autonomy in elderly people, and thus affects their quality of life [3–5]. Nevertheless, only 30 to 48 % of the population benefits from a hearing aid, and 20 to 30 % of them are not satisfied with it [1]. In France, the top reason not to have hearing aids is lack of affordability (78 % of patients said they could not afford hearing aids), followed by the ENT doctor’s opinion and stigma [6].

A few data are available concerning over-the-counter (OTC) hearing aids. Recently, Mc Pherson and colleagues have updated previous studies concerning the electroacoustic characteristics of OTC hearing aids [7]. As in previous studies [8, 9], the authors concluded that the low-cost OTC devices were generally not suitable for the main consumers of these products, i.e. the elderly people [9].

In this context, Tinteo - Personal Sound^©^ society, Meyreuil, France has developed a new OTC hearing aid named TEO First®. This assistive listening device is based on a new sound processing process. This device is a low cost technology (US$250) compared to classical prosthetic devices (between US$1600 and US$2100). The request submitted by Tinteo - Personal Sound^©^ society to the Center for Healthcare Innovation and Uses (CIU-santé) was to assess the value of the TEO First® device in a population of mild to moderate presbycusis patients. We decided to focus our study on elderly patients because of the high prevalence of presbycusis in this population, the potential positive impact of this prosthetic device on their quality of life and because they are the main consumers of these products.

The primary objective of our study was to assess the quantitative earing benefit provided by the use concerning TEO First®. Audiometric evaluation was completed by an assessment concerning the acceptability of this device, its influence on patient’s quality of life and its impact on the project to switch for permanent hearing aid.

## Method

### Location and subjects

This prospective monocentric open label study carried out by the University Hospital of Nice (reference 12-pp-17). The study took place from July 15, 2013, to September 18, 2014. All audiometric assessments were performed by the same audiologist at the IUFC (Institut Universitaire de la Face et du Cou) at the Nice University Hospital (VAT number FR 72 260 600 705). The study was approved by the South Mediterranean V Ethical Review Board (Comité de Protection des Personnes Sud Méditérrannée V, authorization 13.017), as well as by the French Agency for the Safety of Health Products (ANSM, n°ID-RCB 2013-A00149-36). The study was also declared on ClinicalTrials.gov with the number NCT01815788. Patients who agreed to participate gave their written informed consent.

Inclusion criteria were: age over 60 years old with a mild to moderate presbycusis (a bilateral hearing loss in the range between 20 and 50 dB calculated on the average threshold for the tonal frequencies 500, 1000, 2000 and 4000 Hz), wish for a hearing improvement, commitment to use the device daily and sufficient cognitive capacity to use the device. Non-inclusion criteria were: profound hearing loss that cannot be rehabilitated with a prosthetic device, previous use of hearing aid device, previous rejection of hearing aid because of discomfort, aestheticism or local intolerance (ear discharge, perforated eardrum, eczema, mastoiditis sequelae), acute pathology, and legally protected patient. Patients who agreed to participate had to be covered by social insurance. Patients who withdrew consent were excluded from the study. They could also be excluded by the investigator and the study coordinator (mainly for unexpected medical or ethical concerns).

### Recruitment

**Fig. 1:**
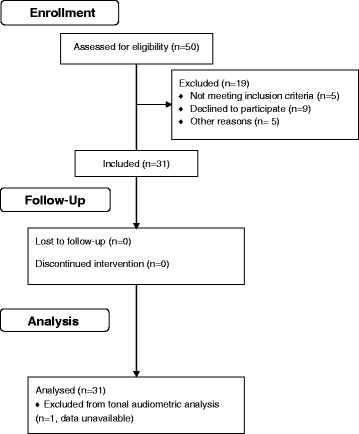
Consort flow diagram

Recruitment was made at the hearing and geriatric clinics of the University Hospital of Nice, and in nursing homes. Fifty patients were screened and thirty one were included (Fig. 1). Screened patients were invited undertake a hearing assessment prior to being included in the study. During this pre-inclusion visit, an explanation of the study and an associated pamphlet were given to the patients. An audiometric evaluation was conducted to diagnose the presbycusis. Patients benefit of an impedance measurement, a pure tone (PTA) and speech (SA) (disyllabic lists) audiometry in silence [10, 11], and also a short form of the Glasgow Hearing Aid Benefit Profile (GHABP) (focus on daily auditory feeling and its impact on quality of life) [12]. The investigators then presented the device and its use. Finally, an acceptability survey (focus on pre-use acceptability of the device) was completed by the patient.

### Materials used for OTC hearing aid

TEO First® is an OTC hearing aid developed by Tinteo - Personal Sound^©^ company. The device is composed of earphones (speakers or headphones 3.5 mm) linked to the electronic system (5*5 cm, 2*2 in.; 32 g, 1.1 Oz). It is sold with software, USB link (for software update), charger, and transport box. The battery is a 350 mAh Li-ion polymer technology battery with a battery life of 10 h and a charging duration of 1.5 h (1000 charging cycles). The amplification range of the device (0 to 22 dB, maximum volume 94 dB) is built on a digital signal processing (DSP) which was initially used for processing sound in the music industry. In this way, the signal is processed in real time, in stereo, on a high fidelity frequency range from 20 Hz to 20 KHz. Sound recording is made by 4 analog Micro-Electro-Mechanical Systems (MEMS) microphones giving a stereophonic effect and improving the signal to noise ratio. The signal is processed via proprietary algorithms. It is composed of: a) a multiband dynamic compression that amplifies soft sounds without amplifying loud sounds; b) filters that allow an improvement of selected frequencies based on presbycusis hearing loss; c) a limiter that avoids transmission of the loudest sounds to users; d) a sound spectral analyses that absorbs disturbing sounds, especially those due to device utilization. The device includes two sound processing modes optimized for calm and noisy environments. It is “pre-fitted” for the average hear-loss of a 65 year old man (calm environment) and the average hear-loss of a 75 year old man (noisy environment). The user can adapt the volume (in 2.5 dB increments, from +0db to +20 dB normalized) and adjust the balance between ears (11 positions are possible). The earphones were standard ones with a foam cover provided to improve fitting. The microphones were located on the device. The patients were advised to clip the device to the belt with the provided clip.

### Experimentation process

Before the first visit, the patients received the informed consent form. They had seven days to make a decision whether to participate in the study. At the beginning of the inclusion visit, the informed consent form was signed in the presence of a physician or the audiologist. After a functional evaluation (Mini Mental State Examination (MMSE) [13]; Instrumental Activities of Daily Living for Elderly (IADL-E) [14]), patients undertook from an audiometric assessment containing acceptable noise level test (ANL) [15], PTA and SA in silence (signal intensity 60 dB) and in noise (signal-to-noise ratio SNR = 10 dB). PTA and SA were evaluated by an audiologist using a calibrated audiometer (Interacoustics AD229b, diagnostic audiometer, Middelfart, Denmark). All measurements were performed in free field in a sound-proof audiometric chamber and the signal was routed to a hi-fi loudspeaker located in front of the patient with white noise coming from two other speakers (at +45° and -45° to the horizontal). The tests were initially carried out without the OCT hearing aid device and then with the OCT hearing aid device (quiet and noisy position each time). After the examination, the device was given a final check, before it was given to the patient with full explanations about the functions of the device. The patient was asked to complete three survey forms after one month of using the device (complete Glasgow Hearing Aid Benefit Profile, acceptability and satisfaction).

The study was ended by a final visit (Day 40) when the physician and the study coordinator received back the surveys and checked the device. All the devices were offered at no cost to the patients at the end of the study. The patients were not informed of this offer until the end of the study.

### Variables studied

The population of the study was characterized by age, sex, level of education, lifestyle, cognitive function (MMSE), autonomy (IADL-E) and ANL. The qualitative benefit to use TEO First® was assessed by the Glasgow Hearing Aid Benefit Profile (GHABP) [12]. Initial acceptability was assessed with a 6-level Likert Scale. The participant was asked six questions about (1) general impression of the TEO First®solution, (2) the level of comfort with technologies in general, (3) the level of comfort with previous use of hearing technologies, (4) the wish to use hearing aids, (5) the expected improvement provided by the TEO First® solution and (6) the reluctance to wear hearing aids continuously. For the final acceptability, we have added 10 questions about the TEO First®solution; (7) the correct use, (8) the ease of use, (9) the agreeableness of use, (10) the usefulness, (11) the satisfaction on quiet setting, (12) the satisfaction on noisy setting, (13) the willingness for daily use, (14) the willingness to use permanent prosthetic device. Question (15) assessed if the duration of one month of use was sufficient to have an objective opinion concerning the device. Question (16) assessed the mean duration of daily use. Excepted for the last question, scoring used the integers 0-5, with zero representing “the worst” concerning the item assess, and five representing “the best” concerning the item assess.

### Statistical analysis

All the data (audiometric tests and survey) were collated on Excel®.

First of all, a descriptive analysis was conducted by a Geriatric Department, Nice University Hospital biostatistician. Results were presented with the number of subjects (N), mean ± standard deviation (SD) for quantitative variables and percentage for qualitative variables. Quantitative data were compared using Student t-test for paired data or Wilcoxon test if t-test conditions were not respected. Qualitative data were compared with Chi square test or Fisher test if Chi square test conditions were not respected. The significance level of all tests was set to 5 %.

Statistical analyses were performed using SPSS® 11.0 (SPSS® Inc, Chicago, USA, IBM© Corporation).

## Results

Thirty one patients were included with 17 women (54.8 %) and a mean age of 78.9 ± 9.7 years (Table 1). At baseline, the voice recognition rate was 58.4 ± 39.2 % in silence and 49.4 ± 39.9 % in noise. There was no difference with or without device concerning ANL test (respectively 5.2 ± 4.5 dB and 6.6 ± 3.9 dB; *p* = 0.071).

**Table 1:** Characteristics of the population

Variable	Men	Women	Total	T-test Men vs women
Age (average ± SD)	76.1 ± 10.2	80.4 ± 8.5	78.3 ± 9.5	ns
Sex (n ; %)	14; 45,2	17; 54,8	31; 100	
MMSE (average ± SD)	25.9 ± 7.8	27.6 ± 10.2	26.8 ± 11.1	ns
IADL-E (average ± SD)	13.5 ± 9.3	16.3 ± 10	15 ± 9.7	ns
Lifestyle (n; %)				ns
Alone	3; 21,4	5; 29,4	8; 25,8	
Married	9; 64,3	3; 17,6	12;38,7	
In family	0,0	1; 5,9	1; 3,2	
Nursing home	2; 14,3	7; 41,2	9; 29	
Other	0	1; 5,9	1; 3,2	
Level of education (n; %)				ns
Under primary school	1; 7,1	0	1; 3,2	
Primary school	2; 14,3	4; 23,5	6; 19,4	
Secondary school	2; 14,3	3; 17,6	5; 16,1	
College	0	3; 17,6	3; 9,7	
University	8; 57,1	5; 29,4	13; 41,9	
Without information	1; 7,1	2; 11,8	3; 9,7	
ANL score (average ± SD)	7,1 ± 3.3	6,2 ± 3.0	6,6 ± 3.9	ns

*SD* standard deviation, *MMSE* Mini Mental State Examination, *IADL-E* Instrumental Activities of Daily Living for Elderly, *ANL* Acceptable Noise Level test

**Fig. 2:**
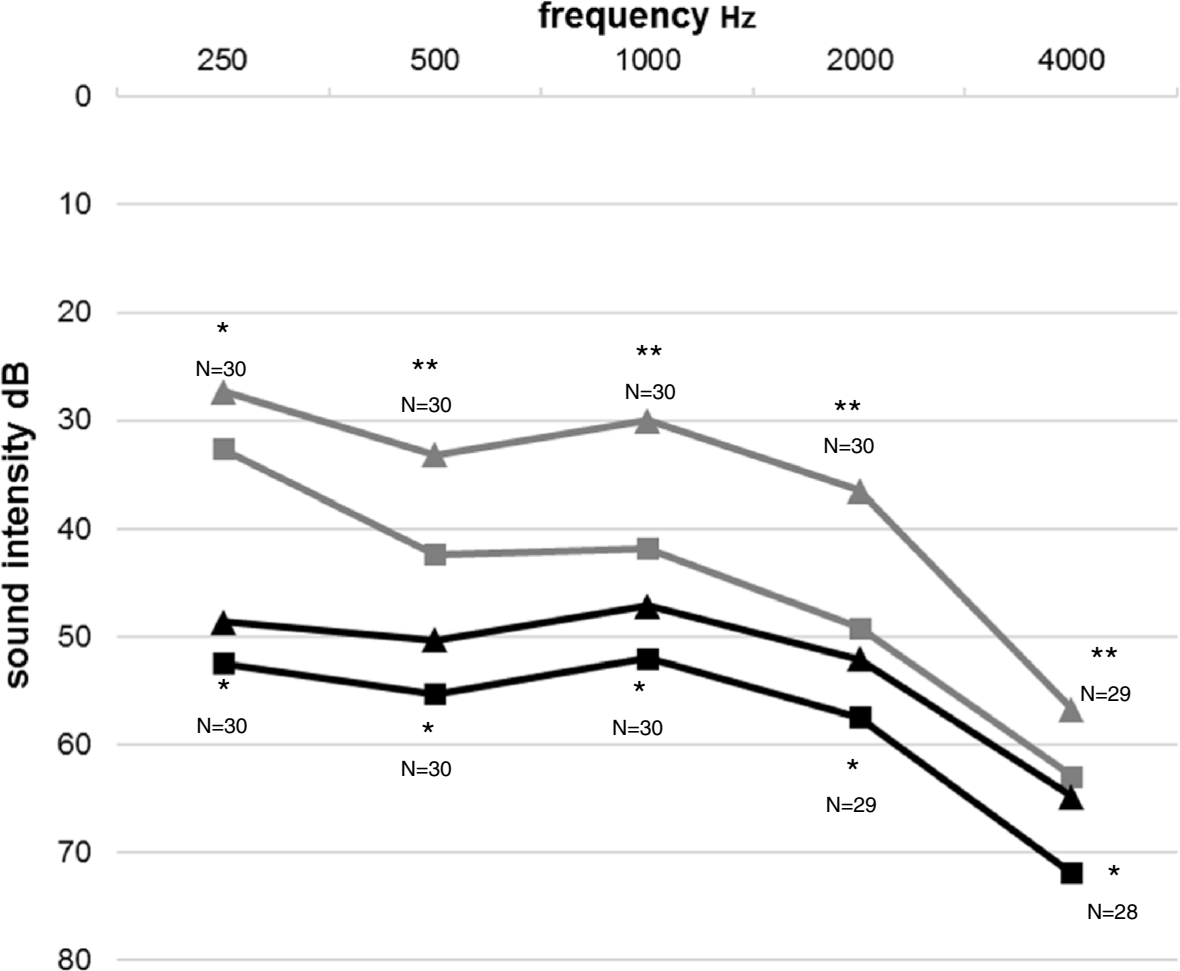
Mean tonal audiometry in silence and in noise (50 dB). * *p* <0.05 ; ** *p* <0.01. grey lines : silent environnement. Black lines : noisy environnement. Triangle : Teo first. Square : no hearing aid

There was an improvement of hearing with TEO First® device in speech and pure tone audiometry, both in silent environment (SA: from 58.4 % at baseline to 81.3 % with TEO First®, +39.2 %, *p* <0.01; PTA: -9.04 dB, *p* <0.05 at 250Hz and *p* <0.01 at 500, 1000, 2000 and 4000 Hz) and in noisy environment (SA: from 49.4 % at baseline to 75.3 % with TEO First®, +47.7 %, *p* <0.01; PTA: -5.23 dB, *p* <0.05) (Fig. 2). After one month of use of the device, quality of life improved with regards to the following parameters: decrease of perceived hearing difficulties during conversation without background noise (-9.6 % *p* = 0.018), in conversation with several people (-16.2 % *p* = 0.0076), decrease of negative emotions while watching TV (-18.5 % *p* = 0.011), during conversation without background noise (-16.5 % *p* = 0.0024), during conversation in noisy background (-17.1 % *p* = 0.027) and during conversation with several people (-20 % *p* = 0.014). Finally, the average daily time of use of the device was 60 min [1st quartile 30 min -3rd quartile 240 min].

Acceptability results were presented with the average ± standard deviation, number of responses (A ± SD, N). The one month trial period was sufficient for the patients to form an opinion concerning the device (4.1 ± 1, *N* = 25). The acceptability of the device was presented in Table 2 and was generally low to moderate : Those using the device reported no significant change in pre‐study reluctance to wear hearing aids daily (median of variation of the reluctance = 0) and in post‐study desirability for continually hearing aids use (2.4 ± 1.8, *N* = 25).

**Table 2:** Results of acceptability surveys

	Score on Likert scales (average ± SD)	N
Pre-test acceptability		
wish to use hearing amplification solution	3.8 ± 0.9	29
sensation of ease with technology	3.7 ± 1.2	27
sensation of ease with previous use of earing device	3.5 ± 1.1	17
overall impression of TEO First® device	4.3 ± 0.8	29
hope in the TEO First® device	4.2 ± 0.7	29
reluctance to wear hearing aids continuously	2.7 ± 1.9	28
Post-test acceptability		
correct use	3 ± 1.4	27
ease of use	3.2 ± 1.6	27
pleasant use	2.2 ± 1.7	27
perceived usefulness	2.4 ± 2	27
satisfaction on quiet setting	2.5 ± 1.7	24
satisfaction on noisy setting	1.8 ± 1.4	21
desire of daily use	2.3 ± 1.9	26

### Adverse event

No adverse events were observed during the study.

## Discussion

To deal with the high prevalence of presbycusis, its direct influence on autonomy in elderly and the low rate of hearing aid usage relative to the prevalence of hearing loss, Tinteo - Personal Sound^©^ company has developed a new OTC hearing aid named TEO First®. The primary objective of our study was to assess the quantitative hearing benefit provided by the use of TEO First®. Audiometric evaluation was completed by an assessment concerning the acceptability of this device, its influence on patient’s quality of life and its impact on the project to switch for permanent hearing aid.

Our study has shown an improvement of hearing with TEO First® especially on speech perception. The use of this device has also improved the patients’ quality of life. Thus, considering that in France low affordability is the top reason not to have hearing aids for 78 % of hearing aid candidates [6] the OTC hearing aid TEO First® appears to be a new effective tool in hearing rehabilitation. This low cost device could potentially facilitate the first step towards hearing rehabilitation. It seems to be a crucial element if we consider the significant consequences of hearing loss, especially the loss of autonomy [3–5].

One of the limitation of our study is the absence of control group. As demonstrated by Munro and colleagues, it is important to control for placebo effects in hearing aid trials and to interpret cautiously any hearing aid trial that did not control for this effect [16, 17]. Nevertheless, the placebo effect described by these study is marginal (+6 % for speech-in-noise performance). Considering the importance of the improvement for the speech comprehension in noisy environment (+47.7 %) in our study, we can consider that the placebo effect does not modify the interpretation concerning our results.

Despite a significant improvement of audiometric parameters with TEO First®, the post-use acceptability was low. This result was consistent with a recent publication indicating a low to moderate interest of bilateral hearing aids for the patients in the four situations explored by the GHABP [18]. This may be due to the very good impression and the great initial expectancy in the performance of TEO First®. Indeed, even if this device has proved its efficacy, it cannot substitute the classical prosthetic devices which perform much better. This gap between initial expectations and real performance can partially explain the low final acceptability.

Moreover, the duration of the study and the small number of participants mean that this study has assessed short term outcomes only. Further studies are needed to confirm these findings and to assess the long term impact of the device.

Nowadays, there are three major obstacles for a massive use of hearing aid. The first is the cost of the devices. In France, the average selling price is 1500 to 1900 euros, with a very high co-payment borne by the patient (91 % after social security reimbursement) [19, 20]. The second is the lack of information and effective screening in target populations with approximatively one third of the elderly people having undiagnosed hearing loss [21]. Indeed, because of the gradual progression of hearing impairment, people may delay or fail to seek professional help. The last is the psychological non-acceptance of the hearing aid devices.

OTC hearing aids could partially remove the first obstacle allowing an easier access to hearing aid devices. Indeed, this category of devices is clearly targeting a low cost market segment compared to digital prosthetic hearing aids. Moreover, OTC hearing aids could also partially solve the problem of lake of information by facilitating access to hearing professionals and therefore improving information as well as screening of deafness.

The satisfaction of the patients with those devices could lead to proposing a digital prosthetic hearing aid to improve hearing rehabilitation. Indeed, to ensure optimum performance of the device, it was necessary to involve the expertise of an audiologist who performed hearing evaluation and adjusted the settings of the device. Nevertheless, our study shows that the wish to use hearing aids continuously was not changed by the use of this OTC hearing aid. Yet, OTC hearing aids could be sufficient hearing rehabilitation devices for certain populations of patients, for example, for financially constrained patients and for those who do not need individual adjustment of digital prosthetic hearing aids. For example, if the patient has difficulties manipulating the device, or in case of cognitive impairment.

## Conclusion

TEO First® is an effective OCT hearing aid that improves the patients’ quality of life. However, our results are short term results and further studies are needed to confirm our findings. Its low cost reduces the financial burden of digital prosthetic hearing aids on the patients. Its use could be a promising way to educate the population and to increase the interest of professional caregivers in hearing loss.

Its use is not expected to replace either the expertise of an ENT professional in the diagnosis or that of an audiologist in the hearing rehabilitation. The device is best positioned as a complementary offer filling the current gap in the field of hearing rehabilitation.

### Abbreviations

ANL, acceptable noise level; ENT, ear nose and throat; GHABP, Glasgow Hearing Aid Benefit Profil; IADL-E, Instrumental Activities of Daily Living for Elderly; MMSE, Mini Mental State Examination; OTC, over the counter; PTA, pure tone analyses; SA, speech analyses
